# The Chicago Medical Examiner

**Published:** 1863-05

**Authors:** 


*The Chicago Medical Examiner*, the editor of which, Prof. Davis, is Chairman of the Committee of Arrangements of the American Medical Association, speaks most encouragingly of the prospects of a large gathering at the next meeting. It thus alludes to the efforts to discourage the meeting:—

"Let no one, outside of Chicago, imagine that the course taken by the *Chicago Medical Journal*, and its senior editor, Prof. D. Brainard, in opposing the meeting of the Association, indicates any division of sentiment or action in the profession here, or that it represents the wishes or feelings of any one here but himself. On the contrary, the profession here are united, and earnestly preparing to give their brethren as cordial and pleasant a reception as they have met with in any other city in our country. Our hotels are of the best character, and amply sufficient to accommodate half a dozen 'Canal Conventions' and Medical Associations at the same time. We have full confidence that the coming annual meeting will be well attended; that its members will transact the legitimate business of the Association with dignity, harmony, and profit; that they will revive and extend past associations and friendships, and by their liberality of sentiment, and their strict adherence to the proper objects of the Association, they will set an example worthy of imitation by all other conventional organizations, whether religious, political, or scientific."

